# Agravamento da Função Renal e Congestão em Pacientes com Insuficiência Cardíaca Aguda: Estudo com Análise Vetorial de Bioimpedância Elétrica (BIVA) e Lipocalina Associada à Gelatinase Neutrofílica (NGAL)

**DOI:** 10.36660/abc.20190465

**Published:** 2021-04-08

**Authors:** Humberto Villacorta, Aline Sterque Villacorta, Leonardo Simões de Castro Villacorta, Analucia Rampazzo Xavier, Salim Kanaan, Felipe Mafort Rohen, Leonardo Dinis Albuquerque, Daniele Dantas Bastilho, Cecília de Oliveira Cudischevitch

**Affiliations:** 1 Universidade Federal Fluminense NiteróiRJ Brasil Universidade Federal Fluminense, Niterói, RJ - Brasil.

**Keywords:** Insuficiência Cardíaca, Insuficiência Renal, Diuréticos/uso terapêutico, Bioimpedância Elétrica, Hemodinâmica, Mortalidade, Hospitalização, Alta do Paciente

## Abstract

**Fundamento:**

O agravamento da função renal (AFR) é frequentemente observado na terapia agressiva com diuréticos para o tratamento de insuficiência cardíaca aguda descompensada (ICAD) e está associado com piores desfechos em alguns estudos.

**Objetivo:**

Avaliar a relação de AFR e congestão na alta hospitalar com ocorrência de eventos (morte cardíaca ou internação por insuficiência cardíaca).

**Métodos:**

Oitenta pacientes com ICAD foram estudados. O AFR foi definido por um aumento absoluto (≥0,5 mg/dL) nos níveis séricos de creatinina a partir dos valores obtidos na admissão. Concentrações de peptídeo natriurético do tipo B (BNP) e lipocalina associada à gelatinase neutrofílica (NGAL) foram medidas na admissão e na alta hospitalar. Congestão foi avaliada na alta utilizando a análise vetorial de bioimpedância elétrica (BIVA). O desfecho primário foi o tempo para o primeiro evento, definido como uma combinação de morte cardíaca ou hospitalização por insuficiência cardíaca. Análise de curva Característica de Operação do Receptor (curva ROC) foi realizada para determinar o ponto de corte de IH mais adequado para predição de eventos. Curvas Kaplan-Meier de sobrevida livre de eventos foram construídas e comparadas usando o teste de log-rank. Modelos de riscos proporcionais de Cox foram usados para investigar a associação com eventos. O critério para se estabelecer significância estatística foi um p<0.05.

**Resultados:**

A idade média foi 60,6 ± 15,0 anos, e 48 (60%) pacientes eram do sexo masculino. A fração de ejeção média foi 35,3±7,8%. O AFR ocorreu em 37,5% da amostra. A creatinina basal associou-se com AFR (p<0,001), mas nem BNP (p=0,35) nem NGAL (p=0,18) na admissão foram preditores de AFR. Usando modelos de riscos proporcionais de Cox, o índice de hidratação na alta, estimado por BIVA, associou-se significativamente com ocorrência de eventos (HR 1,39; IC95% 1,25-1,54, p<0,0001), mas não com AFR (HR 2,14; IC95% 0,62-7,35, p=0,22).

**Conclusão:**

A congestão persistente na alta associou-se com piores desfechos. O AFR parece estar relacionado com alterações hemodinâmicas durante o processo de descongestionamento, mas não com lesões renais.

## Introdução

O agravamento da função renal (AFR) é comumente observado no tratamento agressivo com diuréticos da insuficiência cardíaca aguda descompensada (ICAD) e foi associado a piores desfechos em estudos retrospectivos.^1^ No entanto, resultados contrários foram observados em alguns estudos;^2^^,^^3^ e outros sugerem que a congestão e não o baixo débito cardíaco esteja relacionada com disfunção renal na insuficiência cardíaca (IC).^4^^–^^6^ Além disso, alguns autores mostraram que a presença de congestão persistente na alta hospitalar, independentemente de AFR, está associada com piores desfechos.^7^^,^^8^ Contudo, esses estudos avaliaram congestão com base nos sinais clínicos.

Novas tecnologias podem avaliar água corporal total de maneira mais objetiva por análise de impedância de tecido. Usando análise vetorial de bioimpedância elétrica (BIVA),^9^ nosso grupo, em conjunto com outros centros, já mostrou que quase um terço dos pacientes com ICAD recebem alta hospitalar apresentando congestão persistente, incluindo congestão subclínica, e que esses pacientes apresentam alta mortalidade em 90 dias. A relação de AFR e congestão avaliada por BIVA ainda não foi investigada. O uso dessa tecnologia, ao detectar congestão subclínica, poderia aumentar a acurácia da avaliação de congestão e melhorar a predição de eventos.

Pouco se sabe sobre o mecanismo do AFR após tratamento agressivo com diurético. Não se sabe se o AFR é causado por lesão tubular renal ou se é somente um reflexo de alterações hemodinâmicas que ocorrem durante o tratamento da ICAD. Apesar de a creatinina ser atualmente o biomarcador padrão de função renal, ela tem um aumento tardio após a lesão renal. Além disso, o AFR na ICAD, indicado por um aumento nos níveis de creatinina, pode não refletir lesão renal aguda e não ser prognóstico em todos os pacientes.^2^^,^^3^^,^^7^ A lipocalina associada à gelatinase neutrofílica (NGAL) é um marcador de lesão tubular renal que pode ser medido na urina e no plasma, e que se mostrou mais precisa que a creatinina na predição de lesão renal aguda.^10^^–^^12^

Assim, o objetivo deste estudo foi avaliar a relação entre AFR e congestão persistente na alta hospitalar usando a BIVA na predição de eventos em longo prazo, e avaliar se a lesão tubular renal, avaliada por níveis plasmáticos de NGAL, associa-se com AFR durante o tratamento da ICAD e com o prognóstico após a alta.

## Métodos

### Pacientes

**Figura 1:**
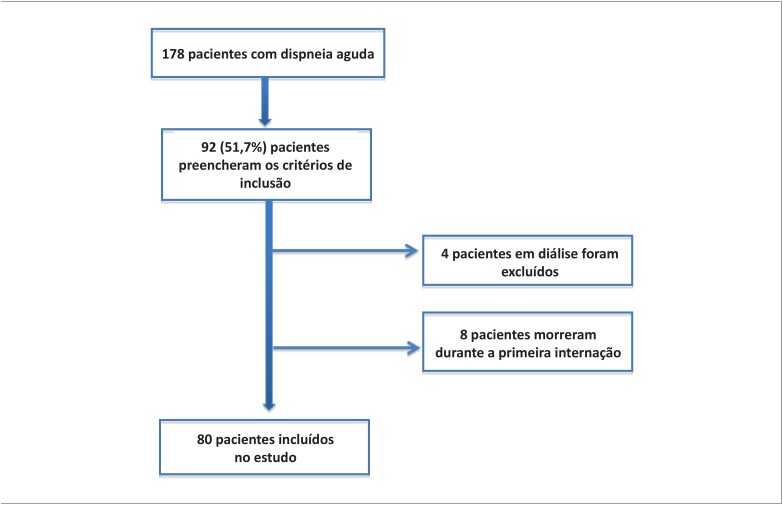
Fluxograma do estudo.

O estudo incluiu 80 pacientes consecutivos com idade ≥ 18 anos, admitidos em um hospital universitário por ICAD. Os critérios de inclusão foram: 1) sinais ou sintomas de ICAD; 2) peptídeo natriurético tipo-B (BNP) > 100 pg/mL na admissão; e 3) fração de ejeção <50% no ecocardiograma. Os critérios de exclusão foram: 1) pacientes com síndrome coronariana aguda como principal causa do episódio atual de ICAD; e 2) pacientes já em diálise antes da inclusão no estudo ou se foi planejado o início da diálise durante a hospitalização. Os pacientes foram tratados seguindo-se as diretrizes de IC, e as decisões sobre o tratamento ficaram a critério dos médicos responsáveis. Os pacientes que foram a óbito antes ou na alta da primeira internação hospitalar foram excluídos das análises. Cada paciente poderia contribuir somente uma vez no banco de dados e, em caso de múltiplas internações, somente a primeira internação que ocorreu durante o período da revisão foi considerada na análise. A Figura 1 ilustra o fluxograma do estudo.

Nosso estudo segue as recomendações do Conselho Nacional de Ética em Pesquisa (CONEP) e foi aprovado pelo comitê de ética de nosso hospital. Consentimento informado foi solicitado e obtido de cada paciente antes de serem incluídos no estudo.

### Medidas

Cada paciente passou por um exame clínico e laboratorial na admissão e durante a internação. Os níveis de creatinina foram avaliados e registrados diariamente desde a admissão até a alta. Um ecocardiograma com Doppler foi realizado durante a internação para avaliar a função sistólica do ventrículo esquerdo (VE).

Os níveis de BNP foram analisados no sangue total utilizando-se o sistema Triage® (Alere Inc, San Diego, CA, EUA), dentro de seis horas após a coleta, na admissão e na alta hospitalar. Os níveis de NGAL foram medidos usando-se o teste Triage NGAL Test (Alere Inc, San Diego, CA, EUA), um imunoensaio que utiliza um cartucho plástico descartável contendo um anticorpo monoclonal marcado com corante fluorescente e NGAL. O teste contém instrumentos de controle, incluindo imunoensaios controle, para se assegurar que o desempenho do teste está adequado e que os reagentes são ativos funcionalmente. Após adição de algumas gotas de sangue ou plasma no poço de amostra, as células são automaticamente separadas do plasma por um filtro. A amostra reage com o anticorpo conjugado ao marcador fluorescente dentro da câmara de reação e desce pela linha de detecção por ação capilar. O conjugado é capturado em zonas discretas de fase sólida resultando em ensaios de ligação específicos para NGAL ou para os antígenos controle. Níveis plasmáticos de NGAL foram analisados na admissão e na alta hospitalar.

**Figura 2:**
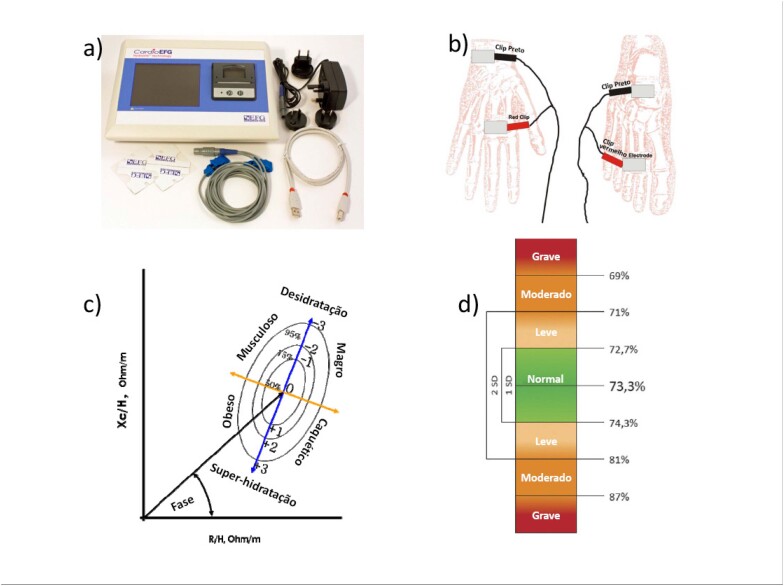
Análise vetorial de bioimpedância elétrica (BIVA). a) Aparelho de BIVA; b) Os eletrodos são posicionados sobre a mão e o pé direitos do paciente; c) Análise vetorial: os sinais são capturados por alguns segundos e, após a análise, de acordo com o ângulo de fase formado pelo vetor, estima-se o grau de hidratação; d) Grau de hidratação de acordo com o índice de hidratação.

O método BIVA foi usado para avaliar água corporal total. Esse método utiliza o programa EFG Renal (Akern, Pontassieve, Florença, Itália) para estimativa dos parâmetros de resistência, reactância, e ângulo de fase. O índice de hidratação (IH) foi então calculado para estimar a quantidade de água corporal total. O intervalo de normalidade para o IH é 72,7% a 74,3%; valores acima desse intervalo indicam congestão, e valores inferiores indicam desidratação. A avaliação por BIVA foi realizada em até 24 horas antes da alta, por um pesquisador independente. Vale destacar que o teste não depende do operador e, portanto, não existe variabilidade interobservador ou intraobservador. O equipamento rejeita o teste em caso de baixa qualidade do sinal. A Figura 2 mostra o aparelho de BIVA e o posicionamento dos eletrodos sobre as mãos e os pés dos pacientes.

## Definições

A ICAD foi definida pela presença de um ou mais sintomas de IC, incluindo dispneia aos esforços, estertores crepitantes, ritmo cardíaco de galope, distensão venosa jugular, ortopneia, dispneia paroxística noturna, uso de mais que dois travesseiros para dormir, fadiga, edema, tosse frequente, tosse com muco ou expectoração sanguinolenta, ou tosse seca ao deitar.

O AFR foi definido como um aumento absoluto nos valores de creatinina sérica ≥0,5 mg/dL a partir dos valores medidos na admissão. Congestão na alta foi definida como um IH >74,3%. Presença de sinais de congestão ao exame físico e IH >74,3% foi considerada congestão subclínica. Para a análise de sobrevida, os pacientes foram divididos em quatro subgrupos, com base na detecção de AFR durante a internação e presença ou não de congestão no momento da alta – ausência de AFR e congestão (sem AFR/sem congestão), presença de AFR na ausência de congestão (AFR/sem congestão), ausência de AFR e congestão (sem AFR/sem congestão), e presença de AFR e de congestão persistente (AFR/congestão). História de doença renal crônica foi definida baseada em um histórico de taxa de filtração glomerular <60mL/min por 1,73m^2^.

### Acompanhamento e Desfechos

Os pacientes foram acompanhados em nosso ambulatório de IC, e as visitas ocorreram em um intervalo de três meses. Se necessário, contatos telefônicos foram realizados para confirmar se o paciente estava vivo ou havia ido a óbito. Não houve perda de seguimento e o período médio de acompanhamento foi de 234 ± 174 dias. O desfecho primário foi o tempo para o primeiro evento, definido como uma combinação de morte cardíaca ou hospitalização por IC.

Hospitalização foi definida como qualquer admissão hospitalar não planejada, que demandou um pernoite. As internações foram classificadas como causadas por IC se causadas por piora dos sintomas de IC, com sinais de sobrecarga de fluidos que requeresse tratamento com furosemida endovenosa.

### Análise Estatística

Os indivíduos foram recrutados por amostragem de conveniência. Os dados são apresentados em média ± desvio padrão (DP), exceto BNP, NGAL, e creatinina, os quais foram apresentados em medianas e intervalos interquartis. As variáveis categóricas foram analisadas pelo teste do qui-quadrado. Para comparação dos dados numéricos, foi usado o teste *t* de Student para amostras independentes ou o teste de Mann-Whitney (não paramétrico). A homogeneidade da variância foi testada pelo teste de Levene. Foram usados métodos não paramétricos, uma vez que algumas variáveis não apresentaram distribuição normal, dada à grade dispersão e rejeição da hipótese de normalidade segundo o teste de Kolmogorov-Smirnov test. Análise de curva Característica de Operação do Receptor (curva ROC) foi realizada par determinar o ponto de corte de IH mais adequado para predição de eventos. Curvas Kaplan-Meier de sobrevida livre de eventos foram construídas e comparadas usando o teste de log-rank. Modelos de riscos proporcionais de Cox foram usados para investigar a associação prospectiva de AFR e congestão persistente com eventos durante o acompanhamento. As variáveis independentes incluídas no modelo foram idade, sexo, IH, AFR, e valores de creatinina, BNP e NGAL na alta hospitalar. O critério para determinar significância estatística foi 5%. As análises foram realizadas pelo programa MedCalc®, versão 14.12.0 (Ostend, Bélgica).

## Resultados

A idade média foi 60,6±15,0 anos e 48 (60%) pacientes eram do sexo masculino. A fração de ejeção do VE média foi 35±7,8%. O AFR ocorreu em 37,5% da amostra. As características dos pacientes com e sem AFR são apresentadas na Tabela 1. As causas da IC foram cardiomiopatia isquêmica em 23 (28,7%), hipertensão em 42 (52,5%), cardiomiopatia em 10 (12,5%), cardiomiopatia alcoólica em 3 (3,7%) e quimioterapia em 2 (2,6%). Os valores de creatinina e IH na admissão foram mais altos e sódio sérico foi mais baixo nos pacientes com AFR. Não houve diferença nos valores de BNP ou de NGAL entre os pacientes com e sem AFR. O período mediano de internação foi de oito dias (intervalo interquartil de 7 a 12 dias). Na alta, a creatinina foi mais alta no grupo com AFR, e o IH foi ligeiramente maior nos pacientes com AFR, diferença estatística entre os grupos.

**Tabela 1:** Características dos pacientes com e sem agravamento da função renal

Variáveis			Com AFR n=30	Sem AFR n=50	Valor de p
Idade (anos)			59,9±17,8	61±13,4	0,75
Sexo masculino			17 (56,7%)	31 (62%)	0,44
Etiologia isquêmica			8 (26,7%)	15 (30%)	0,75
História de diabetes			11 (36,6%)	17 (34%)	0,81
História de hipertensão			22 (73,3%)	34 (68%)	0,61
História de DPOC			5 (16,6%)	8 (16%)	0,94
Fibrilação atrial			6 (20%)	11 (22%)	0,83
Doença renal crônica			13 (43,3%)	16 (32%)	0,31
Frequência cardíaca (bpm)			72,4±8,2	72,7±7,8	0,84
Pressão arterial sistólica (mmHg)			110,3±13,4	110,6±15,5	0,94
Pressão arterial diastólica (mmHg)			69,5±9,8	71,5±9,7	0,37
Fração de ejeção do ventrículo esquerdo (%)			36,7±6	34,5±8,6	0,19
**Características laboratoriais**					
	**Creatinina (mg/dL)**				
		Admissão	1,45 (1,19-1,84)	1,05 (0,91-1,2)	<0,0001
		Pico	2,1 (1,82-2,48)	1,22 (1,13-1,38)	<0,0001
		Alta	1,5 (1,26-1,8)	1,0 (0,87-1,13)	<0,0001
	**NUS (mg/dL)**				
		Admissão	42,4 (23,4-61)	31,4 (18-39,3)	0,007
		Alta	39,6 (21,5-58,4)	30,2 (17,4-36,4)	0,02
	**BNP (pg/mL)**				
		Admissão	806 (531-1276)	667,5 (478-1255)	0,35
		Alta	455 (340-749)	404 (268-661)	0,12
	**NGAL (pg/mL)**				
		Admissão	249,5 (128-539)	216 (92-352)	0,18
		Alta	164,5 (116-286)	190 (98-312)	0,82
	**Sódio sérico (mEq/L)**				
		Admissão	135±4,1	137,6±3,2	0,002
		Alta	137,4±3,9	137,5±3,6	0,93
	**Índice de hidratação (BIVA) %**				
		Admissão	81,3±3,4	78,2±3,2	0,0001
		Alta	77,9±5,8	75,8±4,6	0,08
**Medicamentos na alta**					
		Betabloqueadores	29 (96,6%)	48 (98%)	0,70
		Inibidores de ECA	25 (83,3%)	41 (82%)	0,88
		Bloqueadores de receptor de angiotensina	4 (13,3%)	8 (16%)	0,74
		Espironolactona	17 (56,7%)	31 (62%)	0,64
		Furosemida	29 (96,6%)	47 (94%)	0,60
		Digoxina	2 (6,7%)	4 (8%)	0,83

BIVA: análise vetorial de bioimpedância elétrica; BNP: peptídeo natriurético do tipo B; NUS: nitrogênio ureico no sangue; DPOC: doença pulmonar obstrutiva crônica; NGAL: lipocalina associada à gelatinase neutrofílica; AFR: agravamento da função renal.

O pico mediano de creatinina no grupo com AFR foi 2,1 mg/dL (intervalo interquartil 1,82-2,48 mg/dL). Os valores de BNP diminuíram da admissão à alta tanto no grupo com AFR [806 (531-1276) vs. 455 (340-749) pg/mL, p<0,0001] como no grupo sem AFR [667,5 (478-1255) vs. 404 (268-661) pg/mL, p<0.0001]. O mesmo foi observado para [AFR 249,5 (128-539) vs. 164,5 (116-286) pg/mL, p<0,0001; sem AFR 216 (92-352) vs. 190 (98-312) pg/mL, p=0,0001]. O período médio de internação foi de 8,3±3,1 dias no grupo sem AFR/sem congestão, 11,4±5,3 dias no grupo com AFR/sem congestão, 12,0±4,8 dias no grupo sem AFR/com congestão e 12,5±4,0 dias no grupo com AFR/com congestão (p=0,019). O delta médio da admissão à alta do IH nesses quatro grupos foi, respectivamente 8,4±2,4%, 8,0±2,5%, 5,3±2,6%, e 5,1±2,1% (p=0,0002).

**Figura 3:**
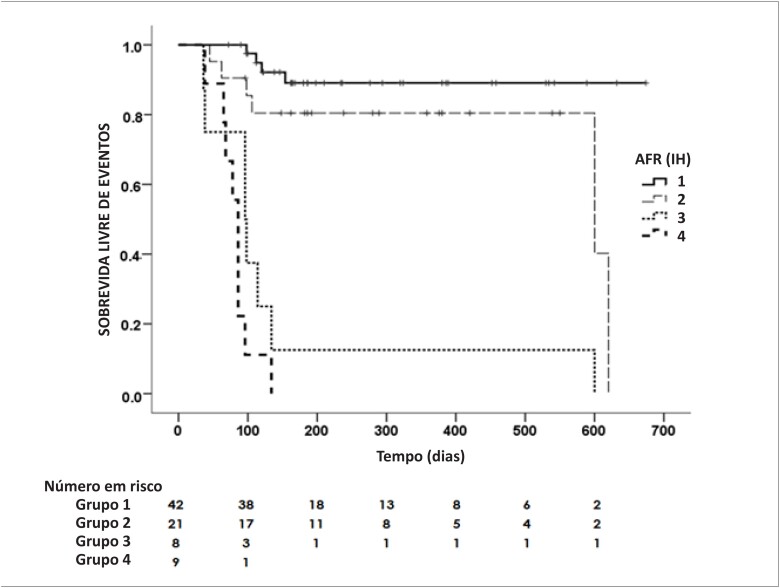
Taxa de sobrevida livre de eventos com base na detecção de agravamento da função renal (AFR) durante a hospitalização, e presença ou não de congestão na alta hospitalar. Grupo = Sem AFR /Sem congestão; 2= AFR/Sem congestão; 3= Sem AFR /Com congestão; e 4= Com AFR/Com congestão (p<0,001); Congestão avaliada pelo índice de hidratação (IH) com análise vetorial de bioimpedância elétrica.

**Figura 4:**
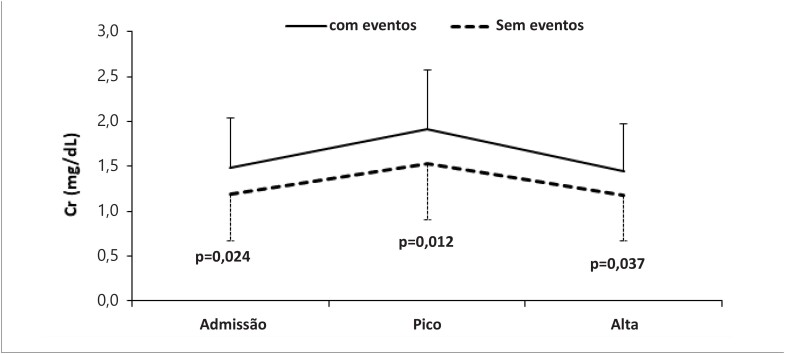
Média de creatinina (Cr) na admissão, pico médio, e média na alta hospitalar em pacientes com e sem eventos. Barras indicam desvio padrão. Valores de p referem-se às comparações entre grupos (Teste t de Student).

Durante o seguimento, foram observados 27 (33,7%) eventos (7 óbitos e 20 internações). As características dos pacientes com e sem eventos são apresentadas na Tabela 2. O número de eventos em cada grupo está descrito na Tabela 3. A Figura 3 mostra as curvas de sobrevida de Kaplan-Meier para os quatro subgrupos de acordo com a presença ou não de AFR e congestão persistente na alta hospitalar. Como observado, pacientes com congestão persistente apresentaram o pior prognóstico. Pacientes com ambas as condições – AFR e congestão persistente – apresentaram uma razão de risco (HR) para morte ou reinternação por IC 9,1 vezes (IC95% 1,41-59,5) à apresentada pelo grupo “AFR/sem congestão” e 27,4 vezes (IC95% 4,5-164,4) à apresentada pelo grupo “sem AFR/sem congestão”. Utilizando o modelo de riscos proporcionais de Cox, o sexo masculino e o IH foram preditores independentes do desfecho primário (Tabela 4). A Figura 4 mostra os valores médios de creatinina na admissão, de pico nos pacientes com e sem eventos. Pacientes com eventos apresentaram valores significativamente mais altos de creatinina em todas as comparações.

**Tabela 2:** Características dos pacientes com e sem eventos

Variáveis			Com eventos n=27	Sem eventos n=53	Valor de p
Idade (anos)			61,6±13,7	60,2±15,9	0,68
Sexo masculino			21 (77,8%)	27 (51%)	0,021
Etiologia isquêmica			9 (33,3%)	14 (26%)	0,47
História de diabetes			10 (37%)	16 (30,2%)	0,46
História de hipertensão			20 (74%)	36 (67,9%)	0,43
História de DPOC			5 (18,5%)	8 (15%)	0,30
Fibrilação atrial			7 (26%)	10 (18,8%)	0,47
Doença renal crônica			10 (37%)	19 (35,8%)	0,91
Frequência cardíaca (bpm)			71,4±8,2	73,3±7,7	0,32
Pressão arterial sistólica (mmHg)			113,8±17,7	108,8±12,6	0,19
Pressão arterial diastólica (mmHg)			71,4±11	70,5±9,1	0,69
Fração de ejeção do ventrículo esquerdo (%)			34,9±7,5	35,6±8	0,68
**Características laboratoriais**					
	**Creatinina (mg/dL)**				
		Admissão	1,29 (1,1-1,76)	1,1 (0,91-1,29)	0,002
		Pico	1,9 (1,40-2,34)	1,3 (1,16-1,75)	0,001
		Alta	1,21 (1,1-1,8)	1,0 (0,88-1,33)	0,003
	**NUS (mg/dL)**				
		Admissão	40,3 (20,4-64)	30,2 (16-35,3)	0,005
		Alta	37,2 (22,3-57,4)	31,4 (15,5-34,2)	0,10
	**BNP (pg/mL)**				
		Admissão	921 (685-1689)	602 (487-964)	0,015
		Alta	580 (390-1210)	377 (277-605)	0,007
	**NGAL (pg/mL)**				
		Admissão	275 (156-478)	187 (100-341)	0,06
		Alta	214 (138-430)	168 (85-312)	0,035
	**Sódio sérico (mEq/L)**				
		Admissão	135±5,1	137,3±3,4	0,018
		Alta	136,4±4,9	138,5±3,2	0,023
	**Índice de hidratação (BIVA) %**				
		Admissão	84,6±3,6	79,2±4,2	<0,0001
		Alta	82,2±4,8	73,7±2,0	<0,0001
		AFR	15 (55,6%)	15 (28,3%)	0,017

BIVA: análise vetorial de bioimpedância elétrica; BNP: peptídeo natriurético do tipo B; NUS: nitrogênio ureico no sangue; DPOC: doença pulmonar obstrutiva crônica; NGAL: lipocalina associada à gelatinase neutrofílica; AFR: agravamento da função renal.

**Tabela 3:** Número de eventos nos quatro grupos de pacientes segundo presença ou não de agravamento da função renal e congestão

Eventos	Sem AFR/sem congestão n=42	Com AFR/sem congestão n=21	Sem AFR/com congestão n=8	Com AFR/Com congestão n=9
Morte	0	3 (14,3%)	2 (25%)	2 (22,2%)
Hospitalização	5 (12%)	3 (14,3%)	6 (75%)	7 (77,7%)
Total	5 (12%)	6 (28,6%)	8 (100%)	9 (100%)

**Tabela 4:** Modelos de riscos proporcionais de Cox para investigar a associação independente de agravamento da função renal e congestão persistente com eventos durante o acompanhamento

Variável	HR	IC95%	Valor p
Idade	1,02	0,98-1,06	0,25
Sexo	3,31	1,04-10,5	0,04
Creatinina	1,08	0,23-4,98	0,91
NGAL	0,99	0,99-1,00	0,51
BNP	0,99	0,99-1,00	0,10
Hidratação*	1,39	1,25-1,54	<0,0001
AFR	2,14	0,62-7,35	0,22

BNP: peptídeo natriurético do tipo B; HR: razão de risco; NGAL: lipocalina associada à gelatinase neutrofílica;

* calculada por análise vetorial de bioimpedância elétrica (BIVA); AFR: agravamento da função renal

## Discussão

O achado mais importante deste estudo foi que a presença de somente AFR durante a hospitalização por IC não está associada com piores desfechos após a alta. Por outro lado, a congestão persistente na alta é um forte preditor de eventos, principalmente em pacientes com AFR durante a internação.

Na admissão, as variáveis associadas com AFR foram creatinina, nitrogênio ureico no sangue, sódio sérico, e IH. A associação entre maior nível de creatinina na admissão e AFR deve-se provavelmente à congestão. Valores baixos de sódio e alto IH corroboram essa hipótese. A congestão dificulta a filtração glomerular e pode resultar em aumento da creatinina.

Estudos iniciais sugeriram que qualquer piora da função renal em pacientes com IC aguda estava relacionada com um pior prognóstico.^1^ No entanto, alguns estudos, com resultados contrários, levaram ao questionamento desse conceito.^2^^,^^3^^,^^7^ Testani et al.,^2^ avaliaram a relação entre hemoconcentração, AFR, e desfechos em pacientes submetidos à terapia agressiva da congestão usando diuréticos durante o tratamento de ICAD. Os autores encontraram uma associação significativa entre hemoconcentração e remoção mais agressiva do fluido e deterioração da função renal. Contudo, pacientes com hemoconcentração apresentaram melhor sobrevida, sugerindo que a terapia agressiva, mesmo no âmbito do AFR, pode ter um efeito positivo na sobrevida.

A relação entre congestão na alta, AFR, e piores desfechos já foi demonstrada em estudos anteriores. No entanto, nesses estudos, o diagnóstico de congestão baseou-se apenas em sinais clínicos.^7^^,^^8^ O dado original em nosso estudo foi o uso de uma avaliação objetiva da congestão por meio da BIVA. Conseguimos demonstrar que, mesmo a congestão subclínica, detectada por essa tecnologia, afeta negativamente a sobrevida e reinternações. Em um estudo prévio,^9^ utilizando BIVA, nós já mostramos que pacientes com ICAD recebem alta com congestão clara ou subclínica, e isso está relacionado a piores desfechos.^9^ Agora, nós confirmamos esse resultado e apresentamos a relação entre congestão e AFR. No presente estudo, um IH >76,5% na alta foi preditivo de eventos. Esse ponto de corte inclui a congestão subclínica, o que pode ter aumentado a sensibilidade para detectar eventos.

Vários estudos demonstraram que a congestão, e não o baixo débito, está associado com AFR.^4^^–^^6^^,^^13^^–^^16^ Em uma análise do banco de dados do ADHERE (*Acute Decompensated Heart Failure National Registry*), de 118 465 admissões por IC, não se demonstrou uma relação entre disfunção sistólica do VE e disfunção renal.^14^ Ainda, em uma análise do banco de dados do estudo ESCAPE (*Evaluation Study of Congestive Heart Failure and Pulmonary Artery Catheterization Effectiveness*), os autores encontraram que, em pacientes com IC descompensada, a função renal não se correlacionou com índice cardíaco, ou resistência vascular sistêmica, e sim com pressão do átrio direito.

A congestão pode levar ao AFR por vários mecanismos.^4^^–^^6^^,^^13^^–^^16^ A congestão venosa renal afeta diretamente a taxa de filtração glomerular.^13^ Além disso, muitas vias abdominais podem levar ao AFR.^13^ Por exemplo, pressão intra-abdominal aumentada, como um marcador de congestão abdominal extrema, está relacionada com disfunção renal em pacientes com IC grave.^13^ Ainda, alterações no baço e no fígado contribuem para congestão e disfunção renal.^13^ Finalmente, hormônios derivados do intestino podem influenciar a homeostase de sódio, enquanto que a entrada de toxinas intestinais no sistema circulatório, consequente a uma barreira intestinal ineficiente secundária à congestão, pode deteriorar ainda mais a função cardíaca e renal.^13^^,^^17^

Com base nesses achados, a terapia agressiva da congestão tem sido proposta como o principal tratamento do AFR na ICAD.^18^^–^^20^ Em um estudo,^18^ um protocolo com intensificação do tratamento com diuréticos em pacientes com AFR e ICAD resultou em maior alteração de peso e maior perda líquida de fluidos após 24 horas em comparação ao tratamento padrão, com uma leve melhor na função renal.^18^

Não encontramos relação entre NGAL na admissão e AFR, ou entre NGAL na alta e desfechos. Nossos resultados estão em acordo com o estudo e Ahmed et al.^21^ que não encontraram nenhuma correlação entre biomarcadores validados de lesão tubular (NGAL, NAG e KIM-1) com AFR em pacientes com ICAD submetidos à terapia agressiva com diuréticos. De fato, aumentos nesses biomarcadores foram paradoxalmente associados com melhor sobrevida.^21^ Esses resultados sugerem que a congestão contribui fortemente para AFR na ICAD e, se a terapia agressiva com diuréticos for realizada, o AFR não tem impacto adverso sobre os desfechos.

No entanto, o presente estudo tem algumas limitações. Primeiro, este é um estudo unicêntrico, e é necessário cautela ao estender esses resultados a outras populações. Segundo, o número de pacientes no presente estudo é relativamente pequeno.

## Conclusão

Em conclusão, utilizando BIVA para avaliar o status de hidratação na alta hospitalar, nós demonstramos que a congestão persistente, e não o AFR, está associada com piores desfechos em pacientes hospitalizados por ICAD. Além disso, encontramos que o AFR parece relacionar-se à congestão e a alterações hemodinâmicas durante o processo de descongestionamento, mas não às lesões tubulares renais, uma vez que não foram encontradas relações entre NGAL, AFR e desfechos.
