# Supplementary material for: Agravamento da Função Renal e Congestão em Pacientes com Insuficiência Cardíaca Aguda: Estudo com Análise Vetorial de Bioimpedância Elétrica (BIVA) e Lipocalina Associada à Gelatinase Neutrofílica (NGAL)
Source: Arq Bras Cardiol. 2021 Apr 8;116(4):715–24. [Article in Portuguese] doi: 10.36660/abc.20190465 (PMC8121398; doi:10.36660/abc.20190465)
Supplement: Translation

# Translation: Worsening Renal Function and Congestion in Patients with Acute Heart Failure: A Study with Bioelectrical Impedance Vector Analysis (BIVA) and Neutrophil Gelatinase-Associated Lipocalin (NGAL)

**Authors:** Humberto Villacorta, Aline Sterque Villacorta, Leonardo Simões de Castro Villacorta, Analucia Rampazzo Xavier, Salim Kanaan, Felipe Mafort Rohen, Leonardo Dinis Albuquerque, Daniele Dantas Bastilho, Cecília de Oliveira Cudischevitch

**Affiliations:** 1 NiteróiRJ Brazil Universidade Federal Fluminense, Niterói, RJ - Brazil.

**Keywords:** Heart Failure, Renal Insufficiency, Diuretics/therapeutic use, Electric Impedance, Hemodynamic, Mortality, Hospitalization, Patient Discharge

## Abstract

**Background:**

Worsening renal function (WRF) is frequently observed in the setting of aggressive diuresis for the treatment of acute decompensated heart failure (ADHF) and is associated with poor outcomes in some studies.

**Objective:**

We sought to assess the relationship of WRF and congestion at discharge with events (cardiac death or heart failure hospitalization).

**Methods:**

Eighty patients with ADHF were studied. WRF was defined by an absolute increase in serum creatinine of ≥0.5 mg/dL from the values measured at the time of admission. B-type natriuretic peptide (BNP) and plasma neutrophil gelatinase-associated lipocalin (NGAL) were measured at admission and at discharge. Congestive state at discharge was assessed using bioelectrical impedance vector analysis (BIVA). Primary endpoint was time to first event defined as a combination of cardiac death or heart failure hospitalization. Receiver operating characteristic (ROC) curve analysis was used to determine the best hydration index cutoff to predict events. Kaplan-Meier event-free survival curves were constructed and compared using the log-rank test. Cox proportional hazards models were used to investigate the association with events. The criterion for determining statistical significance was p<0.05.

**Results:**

Mean age was 60.6±15 years, and 48 (60%) were male. Mean ejection fraction was 35.3±7.8%. WRF occurred in 37.5% of the sample. Baseline creatinine was associated with WRF (p<0.001), but neither admission BNP (p=0.35) nor admission NGAL (p=0.18) was predictor of WRF. Using Cox proportional hazard models, hydration index at discharge calculated with BIVA was significantly associated with events (HR 1.39, 95% CI 1.25-1.54, p<0.0001) but not WRF (HR 2.14, 95% CI 0.62-7.35, p=0.22).

**Conclusion:**

Persistent congestion at discharge was associated with worse outcomes. WRF seems to be related to hemodynamic changes during the decongestion process but not to kidney tubular injuries.

## Introduction

Worsening renal function (WRF) is frequently observed in the setting of aggressive diuresis for the treatment of acute decompensated heart failure (ADHF) and is associated with poor outcomes in retrospective studies.^1^ However, opposite findings have been observed in other studies;^2^^,^^3^ and some studies have suggested that congestion rather than low cardiac output is related to renal dysfunction in heart failure (HF).^4^^–^^6^ Additionally, some authors have shown that persistent congestion at discharge, irrespectively of WRF, is associated with worse outcomes.^7^^,^^8^ However, these studies assessed congestion based on clinical signs.

New technologies can assess total body water more objectively, using tissue impedance analysis. Using bioelectrical impedance vector analysis (BIVA),^9^ our group together with other centers, has previously shown that almost one third of ADHF patients are discharged with persistent congestion, including subclinical congestion, and that these patients have high 90-day mortality. The relationship of WRF and congestion evaluated by BIVA has never been studied. The use of this technology, by detecting subclinical congestion, could increase the accuracy of congestion evaluation and improve the prediction of events.

The mechanism of WRF after aggressive diuresis is poorly understood. There is doubt whether it is a result of renal tubular injury or just a reflection of hemodynamic changes occurring during the treatment of ADHF. Although creatinine is currently the standard biomarker for renal function, it has a delayed increase after kidney injury. Furthermore, WRF in ADHF, indicated by a rise in creatinine, may not reflect acute kidney injury and may not be prognostic in all patients.^2^^,^^3^^,^^7^ Neutrophil gelatinase-associated lipocalin (NGAL) is a kidney tubular injury biomarker that can be measured in urine and plasma, and has been shown to predict acute kidney injury more precisely than creatinine.^10^^–^^12^

Therefore, the aim of this investigation was to assess the relationship of WRF and persistent congestion at discharge as assessed by BIVA in the prediction of long-term events, and to evaluate whether kidney tubular injury, assessed by plasma NGAL, is associated with WRF during the treatment of ADHF and with prognosis after discharge.

## Methods

### Patients

The study comprised 80 consecutive patients aged ≥18 years old, hospitalized in a university hospital for ADHF. The inclusion criteria were: 1) signs or symptoms of ADHF; 2) B-type natriuretic peptide (BNP) >100 pg/mL at admission; and 3) ejection fraction <50% as assessed by echocardiography. Patients were excluded if: 1) acute coronary syndrome as the main cause of current ADHF episode; 2) they were already on dialysis before enrollment or if dialysis initiation was planned during the current hospitalization. Patients were treated according to HF guidelines, and treatment decisions were left to the discretion of the physicians in charge. Patients who died on or before discharge from the initial hospitalization were excluded from the analyses. Each patient could only contribute once to the database, and, in case of multiple admissions, the first hospitalization occurring in the period under review was considered in this analysis. Figure 1 depicts the study flowchart.

**Figure 1:** Study flowchart.

Our study complies with the Brazilian National Council on Ethics in Human Research (CONEP) recommendations and was approved by the Ethics Committee of our hospital. Informed consent was requested and obtained from each patient recruited before entry into the study.

### Measurements

Each patient underwent a complete clinical and laboratory examination at admission and during hospitalization. Serum creatinine levels were assessed and recorded daily from the time of admission until discharge. A Doppler echocardiogram to evaluate the systolic left ventricular (LV) function was performed during hospitalization.

BNP levels were measured in whole blood using the Triage® system (Alere Inc, San Diego, CA, USA), within six hours after collection at admission and at discharge. Plasma NGAL was assessed by the Triage NGAL Test (Alere Inc, San Diego, CA, USA), an immunoassay in a single-use plastic cartridge that contains a fluorescently labeled monoclonal antibody against NGAL labeled with a fluorescent dye and NGAL. There are built-in control features, including control immunoassays, to ensure that the test performs properly and that the reagents are functionally active. Several drops of whole blood or plasma are added to the sample port on the test device, and after this, the cells are automatically separated from the plasma via a filter. The sample reacts with fluorescent antibody conjugates within the reaction chamber and flows down the device detection lane by capillary action. The fluorescent antibody conjugates are captured on discrete solid-phase zones resulting in binding immunoassays that are specific for NGAL or the control antigens. Plasma NGAL of all patients was analyzed at admission and at discharge.

The BIVA method was used to assess total body water. This method utilizes the EFG Renal software (Akern, Pontassieve, Florence, Italy) for estimating the parameters of resistance, reactance, and phase angle. Then, the hydration index (HI) is calculated to estimate total body water. The normal HI range is 72.7% to 74.3%; values above this indicate congestion, and values below this cutoff indicate dehydration. BIVA was performed within 24 h before discharge by an independent investigator. Of note, the test is not operator-dependent and, therefore, there is no intra- or interobserver variability. The machine rejects the test in case of poor signal quality. Figure 2 shows the BIVA machine and the application of electrodes on patients’ hand and foot.

**Figure 2:** Bioelectrical impedance vector analysis (BIVA). a) The BIVA machine; b) the electrodes are placed on the patient’s right hand and foot; c) vector analysis: the signs are captured for a few seconds and after analysis, according to the phase angle formed by the vector, the degree of hydration is estimated; d) degree of hydration according to the hydration index

### Definitions

ADHF was defined as one or more signs or symptoms of HF, including dyspnea on exertion, rales or crackles, gallop heart rhythm, jugular venous distention, orthopnea, paroxysmal nocturnal dyspnea, use of more than two pillows to sleep, fatigue, edema, frequent coughing, a cough that produces mucous or blood-tinged sputum, or a dry cough when lying flat.

WRF was defined by an absolute increase in serum creatinine of ≥0.5 mg/dL from the values measured at the time of admission. Congestion at discharge was defined as HI >74.3%. Patients with no signs of congestion on physical examination and HI >74.3% were considered as having subclinical congestion. For survival analysis, patients were divided into four subgroups, based on the detection of WRF during hospitalization and the presence or not of congestion at the time of discharge, as follows: no WRF and no congestion (no WRF/no congestion), WRF in the absence of congestion (WRF/no congestion), no WRF and congestion (no WRF/no congestion), and presence of both WRF and congestion (WRF/congestion). A history of chronic kidney disease was defined based on a history of an estimated glomerular filtration rate <60 mL/min per 1.73 m^2^.

### Follow-up and Endpoints

Patients were followed-up at our Heart Failure Clinic and visits took place at 3-month intervals. Telephone contacts to assess vital status were made whenever necessary. No patient was lost to follow-up and mean follow-up was 234±174 days. Primary endpoint was time to first event defined as a combination of cardiac death or HF hospitalization.

Hospitalization was defined as any unplanned admission to hospital, which required an overnight stay. Hospitalizations were classified as caused by HF when they were caused by worsening symptoms of HF, with signs of fluid overload, requiring intravenous furosemide treatment.

### Statistical Analysis

Subjects were recruited by convenience sampling. Data are presented as mean ± standard deviation (SD), except for BNP, NGAL, and creatinine, for which median and interquartile ranges are provided. Categorical variables were analyzed using the chi-square test. For comparison of numerical data, Student’s t test for independent samples or the Mann-Whitney test (non-parametric) was used. The homogeneity of the variance was tested by the Levene test. Non-parametric methods were used, since some variables did not present normal distribution, due to the great dispersion and rejection of the normality hypothesis according to the Kolmogorov-Smirnov test. Receiver operating characteristic (ROC) curve analysis was used to determine the best HI cutoff to predict events. Kaplan-Meier event-free survival curves were constructed and compared using the log-rank test. Cox proportional hazards models were used to investigate the prospective association of WRF and persistent congestion with events during follow-up. Independent variables included in the model were age, gender, IH, WRF and creatinine, BNP, and NGAL at discharge. The criterion for determining statistical significance was 5%. Statistical analysis was performed by MedCalc^®^ statistical software, version 14.12.0 (Ostend, Belgium).

## Results

Mean age was 60.6±15.0 years and 48 (60%) were male. Mean left ventricular ejection fraction was 35±7.8%. WRF occurred in 37.5% of the sample. The characteristics of the patients with and without WRF are shown in Table 1. HF etiologies were ischemic cardiomyopathy in 23 (28,7%), hypertension in 42 (52,5%), idiopathic cardiomyopathy in 10 (12,5%), alcoholic cardiomyopathy in 3 (3,7%), and chemotherapy in 2 (2,6%). Creatinine and HI at admission were higher and serum sodium was lower in the WRF group. Neither BNP nor NGAL were statistically different in patients with or without WRF. Median length of stay (LOS) was eight days (interquartile range 7-12 days). At discharge, creatinine was higher in the WRF group, and HI was slightly higher in patients with WRF but this did not reach statistical difference.

**Table 1:** Characteristics of patients with and without worsening renal function

Variables			WRF n=30	No WRF n=50	p value
Age (y)			59.9±17.8	61±13.4	0.75
Male gender			17 (56.7%)	31 (62%)	0.44
Ischemic aetiology			8 (26.7%)	15 (30%)	0.75
History of diabetes			11 (36.6%)	17 (34%)	0.81
History of hypertension			22 (73.3%)	34 (68%)	0.61
History of COPD			5 (16.6%)	8 (16%)	0.94
Atrial fibrillation			6 (20%)	11 (22%)	0.83
Chronic kidney disease			13 (43.3%)	16 (32%)	0.31
Heart rate (bpm)			72.4±8.2	72.7±7.8	0.84
Systolic blood pressure (mmHg)			110.3±13.4	110.6±15.5	0.94
Diastolic blood pressure (mmHg)			69.5±9.8	71.5±9.7	0.37
LV ejection fraction (%)			36.7±6	34.5±8.6	0.19
**Laboratory characteristics**					
	**Creatinine (mg/dL)**					
		Admission	1.45 (1.19-1.84)	1.05 (0.91-1.2)	<0.0001
		Peak	2.1 (1.82-2.48)	1.22 (1.13-1.38)	<0.0001
		Discharge	1.5 (1.26-1.8)	1.0 (0.87-1.13)	<0.0001
	**BUN (mg/dL)**					
		Admission	42.4 (23.4-61)	31.4 (18-39.3)	0.007
		Discharge	39.6 (21.5-58.4)	30.2 (17.4-36.4)	0.02
	**BNP (pg/mL)**					
		Admission	806 (531-1276)	667.5 (478-1255)	0.35
		Discharge	455 (340-749)	404 (268-661)	0.12
	**NGAL (pg/mL)**					
		Admission	249.5 (128-539)	216 (92-352)	0.18
		Discharge	164.5 (116-286)	190 (98-312)	0.82
	**Serum Sodium (mEq/L)**					
		Admission	135±4.1	137.6±3.2	0.002
		Discharge	137.4±3.9	137.5±3.6	0.93
	**Hydration index (BIVA) %**					
		Admission	81.3±3.4	78.2±3.2	0.0001
		Discharge	77.9±5.8	75.8±4.6	0.08
	**Medications at discharge**					
		Betablockers	29 (96.6%)	48 (98%)	0.70
		ACE inhibitors	25 (83.3%)	41 (82%)	0.88
		Angiotensin receptor blockers	4 (13.3%)	8 (16%)	0.74
		Spironolactone	17 (56.7%)	31 (62%)	0.64
		Furosemide	29 (96.6%)	47 (94%)	0.60
		Digoxin	2 (6.7%)	4 (8%)	0.83

BIVA: bioelectrical impedance vector analysis; BNP: B-type natriuretic peptide; BUN: blood urea nitrogen; COPD: chronic obstructive pulmonary disease; LV: left ventricular; NGAL: Neutrophil gelatinase-associated lipocalin; ACE: angiotensin converting enzyme; WRF: worsening renal function.

Median peak creatinine in the WRF group was 2.1 mg/dL (interquartile range 1.82-2.48). BNP dropped from admission to discharge in both WRF [806 (531-1276) vs. 455 (340-749) pg/mL, p<0.0001] and no WRF group [667.5 (478-1255) vs. 404 (268-661) pg/mL, p<0.0001]. NGAL also dropped from admission to discharge in both groups [WRF 249.5 (128-539) vs. 164.5 (116-286) pg/mL, p<0.0001; no WRF 216 (92-352) vs. 190 (98-312) pg/mL, p=0.0001]. Mean LOS was 8.3±3.1 days in the no WRF/ no congestion group, 11.4±5.3 days in WRF/no congestion, 12.0±4.8 days in no WRF/congestion and 12.5±4.0 days in the WRF/congestion group (p=0.019). Mean delta from admission to discharge of HI in these four groups were, respectively, 8.4±2.4%, 8.0±2.5%, 5.3±2.6% and 5.1±2.1% (p=0.0002).

During follow-up, 27 (33,7%) events were observed (7 deaths and 20 hospitalizations). Characteristics of patients with and without events are depicted in Table 2. The number of events in each group is shown in Table 3. Figure 3 shows the Kaplan-Meyer survival curves for the four groups according to the presence of WRF and persistent congestion at discharge. As observed, patients with persistent congestion regardless of WRF during hospitalization had the worst prognosis. Patients with both WRF and persistent congestion had a hazard ratio for death or readmission for HF 9.1 times (95% CI, 1.41-58.5) of that in the ‘WRF/no congestion’ group and 27.4 times (95% CI, 4.5-164.4) of that in the ‘no WRF/no congestion’ group. Using Cox proportional hazards regression analysis, male gender and HI were independent predictors of the primary endpoint (Table 4). Figure 4 shows mean creatinine levels at admission, mean peak, and at discharge in patients with and without events. Patients with events had significantly higher values of creatinine in all comparisons.

**Table 2:** Characteristics of the patients with and without events

Variables			Events n=27	No events n=53	p value
Age (y)			61.6±13.7	60.2±15.9	0.68
Male gender			21 (77.8%)	27 (51%)	0.021
Ischemic aetiology			9 (33.3%)	14 (26%)	0.47
History of diabetes			10 (37%)	16 (30.2%)	0.46
History of hypertension			20 (74%)	36 (67.9%)	0.43
History of COPD			5 (18.5%)	8 (15%)	0.30
Atrial fibrillation			7 (26%)	10 (18.8%)	0.47
Chronic kidney disease			10 (37%)	19 (35.8%)	0.91
Heart rate (bpm)			71.4±8.2	73.3±7.7	0.32
Systolic blood pressure (mmHg)			113.8±17.7	108.8±12.6	0.19
Diastolic blood pressure (mmHg)			71.4±11	70.5±9.1	0.69
LV ejection fraction (%)			34.9±7.5	35.6±8	0.68
**Laboratory characteristics**					
	**Creatinine (mg/dL)**					
		Admission	1.29 (1.1-1.76)	1.1 (0.91-1.29)	0.002
		Peak	1.9 (1.40-2.34)	1.3 (1.16-1.75)	0.001
		Discharge	1.21 (1.1-1.8)	1.0 (0.88-1.33)	0.003
	**BUN (mg/dL)**					
		Admission	40.3 (20.4-64)	30.2 (16-35.3)	0.005
		Discharge	37.2 (22.3-57.4)	31.4 (15.5-34.2)	0.10
	**BNP (pg/mL)**					
		Admission	921 (685-1689)	602 (487-964)	0.015
		Discharge	580 (390-1210)	377 (277-605)	0.007
	**NGAL (pg/mL)**					
		Admission	275 (156-478)	187 (100-341)	0.06
		Discharge	214 (138-430)	168 (85-312)	0.035
	**Serum Sodium (mEq/L)**					
		Admission	135±5.1	137.3±3.4	0.018
		Discharge	136.4±4.9	138.5±3.2	0.023
	**Hydration index (BIVA) %**					
		Admission	84.6±3.6	79.2±4.2	<0.0001
		Discharge	82.2±4.8	73.7±2.0	<0.0001
		WRF	15 (55.6%)	15 (28.3%)	0.017

BIVA: bioelectrical impedance vector analysis; BNP: B-type natriuretic peptide; BUN: blood urea nitrogen; COPD: chronic obstructive pulmonary disease; LV: left ventricle; NGAL: Neutrophil gelatinase-associated lipocalin; WRF: worsening renal function.

**Table 3:** Number of events in the four groups according to the presence or not of worsening of renal function and congestion

Events	No WRF/No congestion n=42	WRF/No congestion n=21	No WRF/Congestion n=8	WRF/Congestion n=9
Death	0	3 (14.3%)	2 (25%)	2 (22.2%)
Hospitalization	5 (12%)	3 (14.3%)	6 (75%)	7 (77.7%)
Total	5 (12%)	6 (28.6%)	8 (100%)	9 (100%)

**Figure 3:** Event-free survival rate based on the detection of WRF during hospitalization and the presence or not of congestion at the time of discharge. 1= No WRF/No congestion; 2= WRF/No congestion; 3= No WRF/Congestion; and 4= WRF/Congestion. Time is depicted as days (p<0.001); Congestion was assessed by hydration index (HI) with bioelectrical impedance vector analysis (BIVA); WRF: worsening renal function.

**Table 4:** Cox proportional hazards models to investigate the independent association of worsening of renal function and persistent congestion with events during follow-up

Variable	HR	95% CI	p value
Age	1.02	0.98-1.06	0.25
Gender	3.31	1.04-10.5	0.04
Creatinine	1.08	0.23-4.98	0.91
NGAL	0.99	0.99-1.00	0.51
BNP	0.99	0.99-1.00	0.10
Hydration*	1.39	1.25-1.54	<0.0001
WRF	2.14	0.62-7.35	0.22

BNP:B-type natriuretic peptide; HR:hazard ratio; NGAL:Neutrophil gelatinase-associated lipocalin;

* estimated by bioelectrical impedance vector analysis (BIVA); WRF: worsening renal function.

**Figure 4:** Mean creatinine (Cr) at admission, peak creatinine, and discharge creatinine in patients with and without events. Bars are standard deviation. P values refer to intergroup comparisons (Students’ t-test).

## Discussion

The main findings of our study are that the presence of WRF alone during HF hospitalization is not associated with worse outcomes after discharge. On the other hand, persistent congestion at discharge is a strong predictor of events, especially in patients with WRF during hospitalization.

At admission, the variables associated with WRF were creatinine, blood urea nitrogen (BUN), serum sodium, and HI. The association between higher creatinine at admission and WRF is probably explained by congestion. Low serum sodium and high HI support this hypothesis. Congestion impairs glomerular filtration and may result in elevation of creatinine.

Initial studies suggested that any worsening of renal function in patients with acute HF was related to a worse prognosis.^1^ However, some studies, with opposite results, led to the questioning of this concept.^2^^,^^3^^,^^7^ Testani et al.^2^ evaluated the relationship of hemoconcentration, WRF, and outcomes in patients submitted to aggressive decongestion during the treatment of ADHF. They found that hemoconcentration was significantly associated with more aggressive fluid removal and deterioration in renal function. However, patients with hemoconcentration had improved survival suggesting that aggressive decongestion, even in the setting of WRF, can positively affect survival.

The relationship of congestion at discharge, WRF, and worse outcomes has already been demonstrated in previous studies. However, the diagnosis of congestion in these studies was based only on clinical signs.^7^^,^^8^ The novel finding in our study is that we used an objective assessment of congestion with BIVA. We were able to demonstrate that even subclinical congestion, as detected with this technology, negatively affects survival and readmissions. Using BIVA we have previously demonstrated that some patients with ADHF are discharged with overt or subclinical congestion and this was related to worse outcomes.^9^ Now we confirm this finding and add information on the relationship of congestion and WRF. In the present study, a HI >76.5% at discharge was predictive of events. This cutoff includes subclinical congestion and may have increased the sensisivity to detect events.

Several studies have shown that congestion, but not low output is associated with WRF.^4^^–^^6^^,^^13^^–^^16^ In an analysis of the ADHERE (Acute Decompensated Heart Failure National Registry) database, of 118,465 HF admissions, a relationship between left ventricular systolic dysfunction and renal impairment could not be demonstrated.^14^ Moreover, an analysis of the ESCAPE (Evaluation Study of Congestive Heart Failure and Pulmonary Artery Catheterization Effectiveness) database, the authors found that in decompensated HF patients, kidney function did not correlate with cardiac index, pulmonary capillary wedge pressure, or systemic vascular resistance, but rather was with right atrial pressure.

Congestion can lead to WRF through several mechanisms.^4^^–^^6^^,^^13^^–^^16^ Kidney venous congestion directly impairs glomerular filtration rate.^13^ Additionally, many abdominal pathways can lead to WRF.^13^ For instance, increased intra-abdominal pressure, as a marker of extreme abdominal congestion, is correlated with renal dysfunction in patients with severe HF.^13^ Moreover, alterations in spleen and liver contribute to congestion and renal dysfunction.^13^ Finally, gut-derived hormones might influence sodium homeostasis, whereas entrance of bowel toxins into the circulatory system, as a result of impaired intestinal barrier function secondary to congestion, might further depress cardiac and renal function.^13^^,^^17^

Based on these findings, aggressive treatment of congestion has been proposed as the mainstay treatment of WRF in the setting of ADHF.^18^^–^^20^ In one study,^18^ a protocol with intensification of diuretic treatment in patients with WRF and ADHF resulted in a greater weight change and greater net fluid loss after 24 hours as compared with standard treatment, with a slight improvement in renal function.^18^

We found no relationship between admission NGAL and WRF nor between discharge NGAL and outcomes. Our results are in accordance with the study by Ahmed et al.^21^ who found no correlations between validated tubular injury biomarkers (NGAL, NAG, and KIM-1) with WRF in patients with ADHF undergoing aggressive diuresis. Of note, increases in such biomarkers were paradoxically associated with improved survival.^21^ Taken together, these findings suggest that congestion is a major contributor to WRF in ADHF, and if aggressive decongestion is promoted, WRF has no adverse impact on outcomes.

However, the present study has some limitations. First, this is a single-center study and caution is advised when extending the findings to other populations. Second, the number of patients in the present study is relatively small.

## Conclusion

In conclusion, using BIVA to assess the hydration state at discharge, we demonstrated that persistent congestion but not WRF is associated with worse outcomes in patients hospitalized for ADHF. Additionally, we found that WRF seems to be related to congestion and to hemodynamic changes during the decongestion process but not to kidney tubular injuries, since no relationship was found between NGAL, WRF, and outcomes.
